# Mesenchymal Stromal Cell-Based Therapy—An Alternative to Arthroplasty for the Treatment of Osteoarthritis? A State of the Art Review of Clinical Trials

**DOI:** 10.3390/jcm9072062

**Published:** 2020-06-30

**Authors:** Tazio Maleitzke, Hisham Elazaly, Christian Festbaum, Christian Eder, Daniel Karczewski, Carsten Perka, Georg N. Duda, Tobias Winkler

**Affiliations:** 1 Center for Musculoskeletal Surgery, Charité—Universitätsmedizin Berlin, Augustenburger Platz 1, 13353 Berlin, Germany; tazio.maleitzke@charite.de (T.M.); christian.festbaum@charite.de (C.F.); christian.eder@charite.de (C.E.); daniel.karczewski@charite.de (D.K.); carsten.perka@charite.de (C.P.); 2 Julius Wolff Institute, Charité—Universitätsmedizin Berlin, Augustenburger Platz 1, 13353 Berlin, Germany; hisham.elazaly@charite.de (H.E.); georg.duda@charite.de (G.N.D.); 3 Berlin Institute of Health (BIH), 10178 Berlin, Germany; 4 Berlin Institute of Health Center for Regenerative Therapies, Charité—Universitätsmedizin Berlin, Augustenburger Platz 1, 13353 Berlin, Germany

**Keywords:** mesenchymal stromal cell, osteoarthritis, regenerative therapy, cell-based therapy, degenerative joint disease

## Abstract

Osteoarthritis (OA) is the most common degenerative joint disorder worldwide and to date no regenerative treatment has been established in clinical practice. This review evaluates the current literature on the clinical translation of mesenchymal stromal cell (MSC)-based therapy in OA management with a focus on safety, outcomes and procedural specifics. PubMed, Cochrane Library and clinicaltrials.gov were searched for clinical studies using MSCs for OA treatment. 290 articles were initially identified and 42 articles of interest, including a total of 1325 patients, remained for further examination. Most of the included studies used adipose tissue-derived MSCs or bone-marrow-derived MSCs to treat patients suffering from knee OA. MSC-based therapy for knee OA appears to be safe and presumably effective in selected parameters. Yet, a direct comparison between studies was difficult due to a pronounced variance regarding methodology, assessed outcomes and evidence levels. Intensive scientific engagement is needed to identify the most effective source and dosage of MSCs for OA treatment in the future. Consent on outcome measures has to be reached and eventually patient sub-populations need to be identified that will profit most from MSC-based treatment for OA.

## 1. Introduction

### 1.1. Osteoarthritis

With a steadily increasing prevalence in past decades, 14 million US individuals are currently estimated to suffer from symptomatic knee OA [1]. A constantly aging society will lead to a dramatic rise in affected patients and concomitant joint replacement surgery in the coming years [2].

Underlying pathomechanisms of OA are versatile and include both, intrinsic and extrinsic factors. Intrinsic factors include age, gender, menopause, genetics, nutrition and bone density, and extrinsic or mechanical factors include body weight, body mass index (BMI), injuries, previous surgeries and joint deformities, which are all directly linked to an increased susceptibility to OA [3,4]. Mostly a combination of these factors and very seldom a single factor alone, leads to a chronic low-grade joint inflammation and a progressive loss of cartilage. Symptoms consist of stern long-term pain, physical disability and a decrease in quality of life [5]. Interestingly, idiopathic OA, unrelated to a specific evident cause or underlying disease, contributes to a high number of OA cases [6].

Self-healing potential in OA is low, which is partly due to the absence of blood vessels and the low metabolic activity of chondrocytes in joint cartilage. Existing treatment strategies for OA generally start with a conservative approach, including physical therapy, exercise and activity modification as well as prescription of analgesics. Non-steroidal anti-inflammatory drugs (NSAIDs) aim at reducing pain and inflammation in the joint. In addition, intra-articular hyaluronic acid (HA)-, glucocorticoid (GC)- or platelet-rich plasma (PRP)-injections are used to achieve temporary analgesia and to reduce inflammation [7]. Continuously progressing OA, which cannot be sufficiently controlled by conservative treatment, can currently only be treated by a few joint-preserving surgical interventions (e.g., osteotomy, joint distraction). Conservative treatment and joint preserving surgery aim at pain reduction and symptom control to prolong the time to unilateral or ultimately total joint replacement surgery, which can effectively replace but not restore joint integrity.

Data from the Norwegian Arthroplasty Register show the ten-year survival rate of primary total knee arthroplasties to be between 89.5% and 95.3% [8]. Further data analyses from the same registry found the ten-year survival rate of primary total knee arthroplasties to be 92.5% in low volume hospitals and 95.5% in high volume hospitals [9]. Despite high overall prosthesis survival rates, implant survival is still highly dependent on the age of patients. Limited prosthesis durability poses still a problem for younger patients and those with impaired bone quality, resulting in increased risks of periprosthetic joint infection, aseptic mechanical failure and subsequent revision surgery [10,11].

Disease modification, potential cartilage repair and regeneration have long been missing in the range of therapeutic options for OA patients. However, the preclinical work has come to a point where cellular therapies to target OA, have become feasible and applicable in first clinical trials over the past decade. A number of clinical trials have recently been performed, that paved the way for a greater acceptance of regenerative cell therapies in OA treatment regimens [12]. Despite a number of unknowns, cell-based treatment created great hopes in OA-affected patients and has become the “poster child” of regenerative medicine.

### 1.2. Mechanisms of Action behind MSCs

Dedicated efforts to decipher and explain regenerative properties and mechanisms of action behind MSCs are part of past and current pre-clinical and clinical studies. Different paradigms and theories have evolved over the years, resulting in two suspected major mechanisms of action on how MSCs could exert their regenerative abilities in OA:

(1) The initially favoured theory, that MSCs mainly differentiate into cells of a specific mesodermal lineage and replace damaged or missing cells has lost its popularity in recent years. However, MSCs seem to share the ability to induce tissue regeneration through the stimulation of local endogenous cells [13].
(2) Today, the major potential of MSCs is seen in their secretion of paracrine factors (“bystander effect”), that allows an immunomodulation of the local pro-inflammatory environment, which plays a key role in cartilage degeneration in OA [14,15]. Whether paracrine effects are long-lasting or merely a “hit and run” phenomenon is currently debated [16].

### 1.3. First MSC Trials and Cartilage Repair

The origin of today’s cell-based therapy concepts dates back to the 1960s, when Friedenstein characterized mesenchymal stem cells or MSCs [17]. MSCs are multipotent precursor cells of mesodermal origin. They are able to differentiate into tissue cells of the mesodermal lineage like chondrocytes, osteoblasts, adipocytes and myocytes. Even a differentiation into neuronal cell types has been described [18]. Due to their differentiation potential, MSCs raised the hope to be exploitable in the treatment of degenerative diseases such as OA through the regeneration of damaged cartilage.

Ashton et al. were the first to report of the chondrogenic potential of MSCs in vivo in 1980 [19]. 30 years later, Mokbel et al. showed that the repair of damaged articular cartilage tissue happened through homing, engraftment and production of cartilage matrix by MSCs [20]. Further animal experiments using MSCs for the treatment of OA followed, demonstrating reduced articular cartilage degeneration, osteophytic remodelling, and subchondral sclerosis after intra-articular injection of MSCs [13,21,22].

Early in vitro studies demonstrated the capacity of MSCs to form cartilage-like tissue, evident by the production of type II collagen and hypertrophic chondrocytes after growth-factor stimulation [23]. Yet, the quality of tissue-engineered cartilage was reported to be compromised, indicated by low collagen contents with a presumably negative impact on the load-carrying properties of cartilage [24]. New cell-carrier scaffolds, made from natural cartilage extra-cellular matrix, promoted proliferation and chondrogenic differentiation of bone-marrow-derived MSCs (BMMSCs) without outside stimulations in an in vivo animal model [25].

A 2002 study published by Wakitani et al. marked one of the first OA trials of BMMSCs used in humans. Twenty four patients suffering from knee OA were either treated with a high tibial osteotomy (HTO) combined with intra-articular autologous cultured BMMSC injections or with an HTO alone as a control group. At the 16-month follow-up, pain scores, function and muscle strength had significantly improved in the treatment compared to the control group [26].

With the rise of this new cellular-based therapy, concerns about the quality of autologous MSCs from patients suffering from OA arose. In 2002 Murphy et al. described a compromised ability of MSCs from OA patients to differentiate into chondrocytes [27]. A more recent study however found that MSCs from OA patients did in fact have the same chondrogenic differentiation potential as age-matched patients with femoral fractures [28]. These contradicting results may be explained by the fact that the compared patient collectives in the study by Murphy et al. [27] were not strictly age-matched and an isolated impact of age may not be disregarded, as MSCs were described to lose their chondrogenic differentiation potential with age [29,30,31]. The authors further discussed whether the reduced differentiation potential of progenitor cells may even be a cause for OA, rather than a result of it [27].

An increased chondrocyte proliferation was observed when chondrocytes were co-cultured with MSCs and proliferation was further associated with the ability to synthesize cartilaginous extracellular matrix in vitro [32,33]. In turn, anti-proliferative effects have been described, when MSCs were challenged with “negative” signals, like interferon-γ or bacterial stimuli like lipopolysaccharide [22,34].

### 1.4. OA as an Inflammatory Disease and Immunomodulatory Properties of MSCs

For a long time, OA was classified as a non-inflammatory “wear and tear” disease. This changed through the works of Goldring and Scanzello et al., who helped to categorize OA as a stress-induced and pro-inflammatory disease in 2011 [35,36].

Numerous studies followed, concluding the same and showing a direct role of pro-inflammatory factors, such as tumor necrosis factor (TNF)-α and interleukin (IL)-1β in OA pathogenesis [37,38]. Since then inflammation and synovitis have been fundamentally included in the theoretical framework of the OA pathomechanism and are also considered targets for new treatment modalities such as MSC-based therapy [16,39]. In addition to the differentiation potential of MSCs towards chondrocytes and the recruitment of neighbouring progenitor cells, it was proven that MSCs have potent anti-inflammatory as well as immunomodulatory properties [40,41].

Immunomodulatory effects of MSCs were reported for adipose tissue-derived MSCs (ATMSCs) from different adipose tissues (abdominal fat, infrapatellar Hoffa fad pad, subcutaneous hip fat), as they show a reduction of inflammatory factors like TNF-α, IL-1β and CCL3/macrophage inflammatory protein-α, when co-cultured with chondrocytes and synoviocytes from OA patients [42]. The immunomodulatory effects of ATMSCs and BMMSCs show some similarities, as both cell types reduce T-cell numbers and subsequent pro-inflammatory cytokines [43,44].

The mechanism behind the regulation of local inflammatory environments, is in part explained by the secretion of growth factors, chemokines, cytokines, micro-RNAs and extracellular vesicles (EVs) by MSCs. MSC-secreted factors affect macrophages, dendritic cells, T- and B-cells, neutrophils and natural killer cells [45]. Further, ATMSCs and BMMSCs decrease antibody production and B-cell chemotaxis by inhibition of B-cell proliferation through cell-cycle arrests and apoptosis in a T-cell independent manner [46,47].

Keeping in mind that inflammation plays a key role in osteoarthritic joints, immunomodulatory properties have been used to explain partial joint tissue healing through MSCs. The deployment of a suitable environment for tissue regeneration by MSCs may therefore either be achieved by direct secretion of bioactive materials or by controlling and containing cytokine and growth factor production from effector cells [44]. Anti-inflammatory and immunomodulatory effects of MSCs can further be modulated and enhanced by ex vivo “priming” of MSCs with cytokines, hypoxia, chemical agents, biomaterials, and receptor challenging. A comprehensive literature overview regarding priming approaches for MSCs was recently published by Noronha NC et al. [48] and a summary of growth factors, chemokines, cytokines, micro-RNAs and EVs, secreted by MSCs was provided by Lin et al. [45].

### 1.5. Oncological Safety Profile of MSCs

Due to the high proliferative capacities of MSCs and the potential of tumour-derived cytokines to attract MSCs to tumour sites, oncological side effects must be discussed when assessing the safety profiles of cell-based therapies. MSCs have shown to exert potential tumorigenicity in several murine and in vitro models, yet no novel cancers or cancer recurrences have been diagnosed in clinical trials to date, that would originate from administered MSCs as summarized in recent reviews by Lee et al. [49] and Ridge et al. [50].

Toyserkani et al. conducted a concise systematic review about the safety of ATMSCs in 2017, assessing 70 studies with over 1400 patients, who received ATMSCs for various conditions. Thromboembolic complications were registered for the systemic application of MSCs, yet the local treatment appeared to be safe, especially regarding oncological side effects. One case of breast cancer recurrence was identified in 121 patients with a known history of previous breast cancer. Although the authors came to the conclusion that ATMSCs have a favourable oncological safety profile, they stressed the low quality of reporting of adverse events (AEs) and the short follow-up period of 12 months [51]. Standardized reporting systems for AEs and long-term follow-ups of several years are therefore needed to fully evaluate the oncological safety of MSC-based therapy in the future.

### 1.6. Preparation of MSCs

MSCs can be harvested from various tissues of mesenchymal origin. These include e. g. bone-marrow, adipose tissue, synovial fluid, skeletal muscle and the placenta. MSC-based therapy for OA follows the basic principle of a local application of harvested cells into the affected joint, after ex vivo isolation, concentration or cell expansion. Delivery of MSCs is either achieved through an intra-articular injection or an arthroscopically assisted implantation of MSCs. The distinction between autologous and allogenic sources stems from the origin of MSCs. Allogenic cells are harvested from a suitable donor, whereas autologous cells originate from the patients themselves. In general, autologous MSCs are often seen as the safe choice, as concerns about unwanted immune-responses or transplant rejections can be disregarded [52]. The complex autologous MSC preparation procedure that may include cell culture and expansion, makes autologous solutions however a more complicated and expensive method opposed to allogenic alternatives. Allogenic MSCs are cheaper and logistically more convenient, as they can be obtained as an off-the-shelf product [53].

In the context of ex vivo expansion, xenogenic elements (e.g., remains of fetal bovine serum) should, if possible, be replaced by humanized material or eliminated as far as possible to reduce post-transplantation risks such as transmission of microbes (viruses, bacteria, prions), cytotoxicity, uncontrolled and unpredictable growth patterns as well as the theoretical risk of anaphylactic reactions [54].

Further, some patients’ comorbidities (e.g., anaemia, severe osteoporosis) do not allow harvesting of autologous MSCs and certain underlying autoimmune conditions like rheumatoid arthritis [55,56] and type 2 diabetes [57], may impair the immunosuppressive capacities of MSCs, which in those cases leaves allogenic MSC-based therapies as the more feasible choice. Generally, three types of altered autologous MSC products can be distinguished:

(1) MSC concentrates are the product of harvested fluids or tissues, which have been concentrated in order to increase the number of MSCs per unit of applied suspension. Bone-marrow aspirate concentrates (BMAC) include high concentrations of growth factors and high levels of anti-inflammatory IL-1 receptor antagonist next to MSCs [58].
(2) Culture-expanded MSCs can be administered without scaffolds or seeded onto scaffolds after having been isolated and cultured. Traditionally two-dimensional (2-D) plastic culture plates are in use for cell expansion, but three-dimensional (3-D) techniques have evolved in recent years. 3-D cultures are able to mimic in vivo conditions and provide high density and expansion potential [59]. New additions like highly elastic culture dishes, automatically adapting the dishes’ surface to growing cell numbers, optimize the expansion process before MSCs are ready for application [60]. Increased complexity and non-standardized expansion protocols, alongside higher costs should be taken into consideration, when applying 3-D expansion techniques. However, reports about increased immunomodulatory and chondrogenic potential of MSCs, cultured in 3-D compared to conventional 2-D cultures, point towards promising technical developments in the field of tissue engineering for OA treatment [61,62].
(3) The stromal vascular fraction (SVF) is a combination of ATMSCs, endothelial cells, growth factors, precursor cells, macrophages, t-regulatory cells, lymphocytes and others. The SVF is derived from lipoaspirates by mechanical or enzymatic isolation [63]. The SVF can be injected into the joint, after a purification process, often within the same visit, as there is no need for expansion or culturing of cells.

As with many novel therapeutic strategies, a standard is not yet available and different approaches are present in the current literature regarding cell origin, harvesting technique, cell dose, culturing and application. Moreover, controversy prevails regarding outcome measurements and follow-up times in current study designs. In this review we analysed the relevant clinical literature on MSC-based treatment for OA with special regards to safety and efficacy. As the field of regenerative medicine and especially the use of MSCs in degenerative joint disorders has become more and more relevant for the medical and public community, we hope to shed some light on this valuable and fast-evolving treatment concept.

## 2. Methods

First step: A literature search was performed to identify all relevant articles, involving MSC-based treatment for OA. Pubmed (MEDLINE), The Cochrane Library and clincialtrials.gov were searched until the 13 of April 2020. For Pubmed and The Cochrane Library searches, MeSH (Medical Subject Headings)-terms were used as shown below.

(1) PubMed MeSH search terms: “mesenchymal stem cell transplantation” [Mesh] AND “osteoarthritis” [Mesh]: 290 results
(2) Cochrane Library MeSH search terms: #1 MeSH (“mesenchymal stem cell transplantation”) explode all trees AND #2 MeSH (“osteoarthritis”) explode all trees: 16 results
(3) ClinicalTrials.gov search terms: “mesenchymal stem cells” OR “mesenchymal stromal cells” AND “osteoarthritis”: 96 results

Second step: Relevant studies were identified through abstract information and then included or excluded after full-text evaluation.

We included all clinical studies, that used MSCs to treat OA in humans, with a recruitment of at least seven participants. Articles from any country were included but limited to those published in English language. We excluded any preclinical studies, case reports, review articles and studies addressing isolated, focal chondral defects not clearly associated with OA. Further excluded were all articles older than ten years, to ensure actuality of this review.

Third step: Reference lists of included articles were scanned regarding potentially missed studies. Missed studies were then identified and added by hand.

Fourth step: Included articles were analysed regarding number of patients, follow-ups, treatment details, outcome measures, study type, control arm and site of OA (see Table 1).

**Figure 1:**
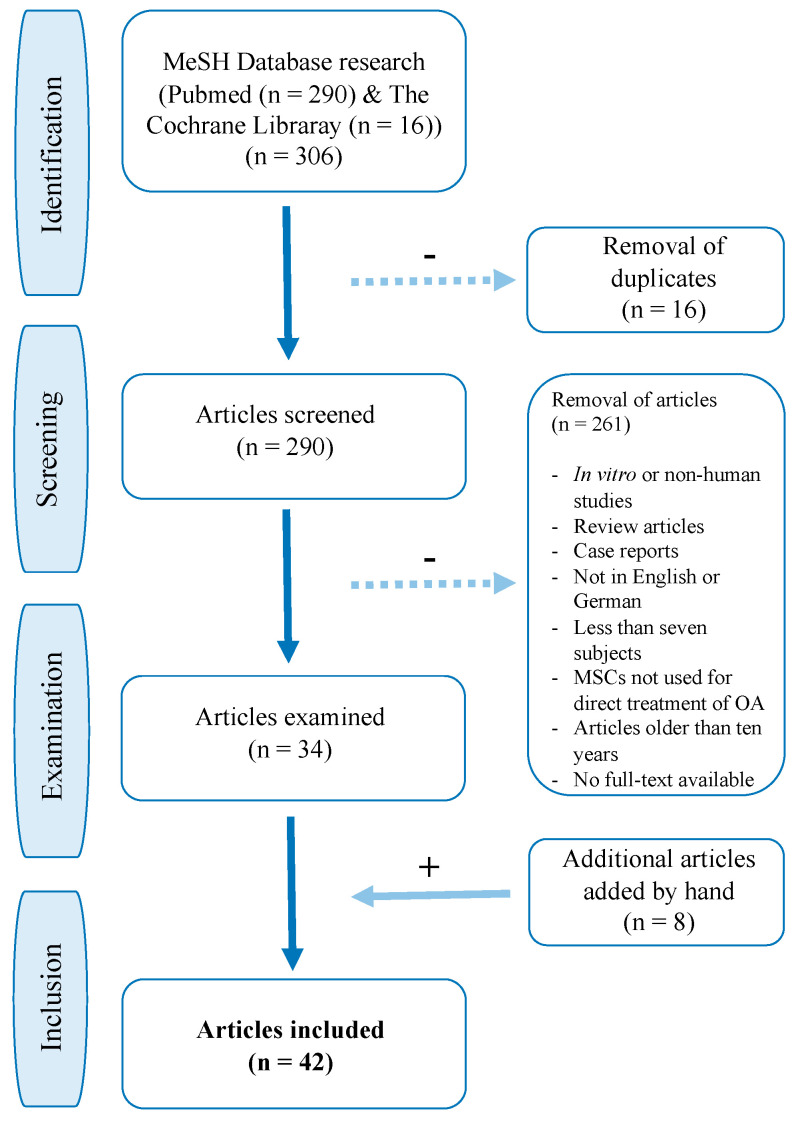
Flow chart of PubMed and The Cochrane Library study selection.

The initial literature search of PubMed yielded a total of 290 articles. The search of The Cochrane Library database yielded 16 results, of which all had already been covered by the previous Pubmed search. After removal of duplicates 290 articles were scanned and full-text evaluation followed. 34 articles were identified as relevant to this review. The reference lists of the 34 selected articles were scanned regarding missed studies and eight studies were added by hand. A total of 42 studies, including 1325 patients, were examined in detail (see Figure 1 and Table 2).

As of April 13th 2020, the search on clinicaltrials.gov yielded 96 results. 25 trials were currently recruiting OA patients for MSC treatment (see Table 1) and two additional trials were currently recruiting, but not addressing MSC treatment in OA directly (acetabular labrum, “synovial brushing” study). 34 trials were already completed, 11 were not yet recruiting and 24 were either “withdrawn”, “unknown”, “suspended”, “terminated” or “active, not recruiting”.

## 3. Study Designs and Route of Cell Delivery

A total number of 42 clinical studies including 1325 OA patients were evaluated regarding study type, methods, techniques and outcome measures. The majority of studies focused on OA of the knee [63,64,66,67,68,69,70,71,72,74,75,76,77,78,79,80,81,82,83,84,85,86,87,89,90,91,92,93,94,95,96,97,98,99,100,101,102,103,104,105], while two studies treated OA of the hip and ankle [73,88]. This may be due to the fact that knee OA has a high prevalence amongst all forms of OA with an estimated lifetime risk of symptomatic manifestation of 40% in men and 47% in women [106,107] and a rather safe and well-described protocol for intra-articular knee-injections [108].

In this review we identified six randomized, double-blinded, placebo-controlled trials, including one follow-up study [64,65,66,78,105,109], eight randomized controlled trials [67,68,69,79,81,82,83,103] and two prospective, randomized cohort studies [70,84]. Most studies were however prospective uncontrolled clinical trials, comparative matched pair analyses, retrospective comparative studies or retrospective case series and an inter-study comparison was difficult due to the variance in study design.

Studies varied further regarding protocols for intra-articular MSC delivery route. Local application was either achieved through simple intra-articular injections of MSCs mixed with saline [64,66,99,103,104,110] or PRP [69,70,81,87,89,93,96,97]. Implantation of MSCs was further supported by allogenic cartilage [84], or the use of fibrin glue scaffolds [85,91,94]. Park et al. used drill holes [104] and other groups combined MSC applications with surgical correction operations like HTO for knee OA [81,84,86] or lateral sliding calcaneal osteotomy for ankle OA [88].

## 4. Safety

Although not all of the included studies evaluated the safety of MSC-based treatment for OA, all reported treatment-related serious adverse events (SAEs) and AEs were described to have subsided without sequelae. Treatment-related AEs included either injection site complications like local knee pain and swelling or harvesting site complications like low back pain after iliac bone harvesting and haematoma after liposuction.

Gupta et al. reported one treatment-related SAE (synovial effusion) and nine treatment-related AEs in a study of 60 patients with knee OA after intra-articular injections of allogenic BMMSCs. No sequelae of the SAE were reported [78]. Bastos et al. reported two treatment-related SAEs (intense knee pain) and eight treatment-related AEs in 18 knee OA patients, treated with autologous BMMSC injections. SAEs were treated with analgesics and resolved without lasting impairments [70].

Mild to severe AEs were reported in 80–100% of patients receiving either single or repeated ATMSC injections for knee OA in a trial by Freitag et al. No significant difference was observed between treatment groups. Discomfort and swelling at the injection site were the most commonly reported AEs [83].

Soler et al. reported of 14 mild and two moderate treatment-related AEs ranging from arthralgia and joint swelling to knee locking and back pain in a study of 15 knee OA patients treated with autologous BMMSC injections [74]. Orozco et al. reported transient and mild injection site pain for a few days in 50% of patients in a study treating 12 knee OA patients with autologous BMMSCs [76]. Song et al. reported 20 treatment-related AEs in 18 patients, receiving different intra-articular cell doses of autologous ATMSCs for OA of the knee. Transient pain and joint swelling occurred equally distributed among groups and were all spontaneously relieved [98]. Chahal et al. treated 12 patients with BMMSCs for knee OA and reported of four patients with minor transient AEs (local pain and swelling), which all subsided without intervention [72].

None of the authors reported any lasting impairments resulting from treatment-related SAEs or AEs. All reported cases resolved within a few days and no patient had to terminate any of the trials preliminary due to safety issues. Moreover, reported AEs were similar to those previously reported during clinical trials, evaluating intra-articular injections of HA or GC [111,112]. It can therefore be assumed that treatment-related AEs are of procedural nature and that intra-articular MSC injections are safe, regardless of tissue origin and applied cell dose. Long-term follow up studies of seven and four years, conducted by Park et al. [104] and Lamo-Espinosa et al. [15] reported no further SAEs and AEs that persisted over the acute post-injection phase.

## 5. Duration of Therapeutic Effects

Preferably, an ideal treatment should improve symptoms for a long duration, halt disease progression and ultimately heal the patient’s condition. Unfortunately, this is rarely the case and an amelioration of symptoms for as long as possible is a more realistic goal for most therapies treating degenerative joint diseases. Taking a look at scheduled follow-ups of included studies, most studies followed-up on their patients for six to 24 months [64,66,67,68,69,70,71,72,74,75,76,77,78,79,80,82,83,87,90,94,95,96,98,99,100,101,102,103,105].

Almost all included studies recorded clinical improvements and cartilage regeneration for the first 12 months after the treatment. Studies with longer follow-up periods reported further improvements over time [85,86,88,89,90,95], plateau phases with maintained clinical improvements [100,104], or deterioration of previous improvements after 30 months [73]. A four-year follow-up study, conducted by Lamo-Espinosa et al., showed clinical and functional improvements four years after a single intra-articular injection of BMMSCs [65]. Larger randomized controlled trials with extended follow-up visits will be needed to gain further insights into the question of how long locally injected MSCs can exert their effects on the joint. So far, relevant clinical improvements have been recorded for as long as six to 12 months in most published studies.

## 6. Quality of Life and Mental Health

The Quality of Life Short Form-36 (SF-36) questionnaire evaluates quality of life, using eight sub-scores, concerned with the physical and mental status of the patient. It is widely used to evaluate therapy success with a focus on the patient’s physical and emotional state during or after an intervention. Interestingly, while most evaluated clinical (e.g., pain visual analogue scale (VAS) score) and structural (e.g., Whole-Organ Magnetic Resonance Imaging Score (WORMS)) parameters of included studies improved over time, results of the SF-36 did often not correlate [74,76,101,103]. The SF-36 questionnaire is often criticized for being too generic and less sensitive than for example the Western Ontario and McMaster Universities OA Index (WOMAC) score, which was specifically developed to assess lower extremity arthritis [113]. The SF-36 questionnaire should therefore be interpreted with caution and may be replaced if possible, by more specific alternatives.

## 7. Radiological and Arthroscopic Outcome Evaluations

To quantify newly-formed cartilage following MSC-based therapies, MRI studies [67,72,74,79,80,82,83,97,103,105,114] or second-look arthroscopy interventions [81,84,85,86,88,89,92,93,99,100,104] can be performed to analyse structural improvements through MSC-based treatment.

MSC-induced cartilage improvements, visible in MRI, are an ongoing topic of debate and contradicting results are continuously reported. While some authors demonstrate cartilage regeneration in accordance with clinical improvements [76,77,82,87,97,99], others could not confirm changes in cartilage structure, although clinical improvements were observed [95,103,115].

In 2008 Centeno et al. reported one of the first intra-articular injections of autologous BMMSCs in a patient with OA of the knee. The group found that six months after treatment the patient had already shown statistically significant growth of cartilage and meniscus in the conducted MRI [114]. Soler et al. and Vega et al. could both confirm improved cartilage quality at the 12-month follow-up for knee OA patients, treated with BMMSCs [74,79]. Wong et al. showed a significantly improved Magnetic Resonance Observation of Cartilage Repair Tissue (MOCART) score one year after HTO, microfracturing and intra-articular injection of autologous BMMSCs mixed with HA in 28 knee OA patients compared to the control group, comprised of 28 OA patients who underwent the same procedure without MSC injection [67]. In 2013, Koh et al. reported that improved MRI scores were positively correlated with the number of injected MSCs [97].

Pintal et al. treated 19 knee OA patients with autologous ATMSCs and although they observed a significant improvement in clinical scores at the six- and 12-month follow-up, no significant differences in cartilage constitution were observed in the conduced MRI [96]. Gupta et al. treated 60 knee OA patients with varying doses of allogenic BMMSCs against a control group. While pain VAS and clinical scores improved in all treatment groups, no cartilage changes were detected [78]. In accordance, Matas et al. found significant pain and functional improvements in knee OA patients, treated with UCMSCs, yet no structural changes were evident in conducted MRI [103].

As an alternative more invasive outcome measure, arthroscopic evaluations can be conducted, which are mostly performed as a second-look intervention at a scheduled follow-up.

In 2018 Kim and Koh performed a comparative matched-pair analysis, comparing outcomes of HTO-treated with HTO + SVF-treated knee OA patients. The authors reported significant cartilage improvements in the latter, according to ICRS grades, assessed 12 months after the intervention. ICRS grades correlated further with clinical outcome measures [86].

Koh et al. compared HTO + PRP + autologous ATMSC-treated (*n* = 21) with HTO + PRP-treated knee OA patients (*n* = 23) and second-look arthroscopy after a mean of 19.8 months showed significantly more cartilage healing in the HTO + PRP + ATMSC group [81]. According to the Kanamiya grading system [116], 50% of patients in the HTO + PRP + MSC group showed partial or fibrocartilage cover, whereas this was only shown for 10% in the control group [81].

Most of the aforementioned studies need to be interpreted with caution as they showed a low level of evidence. Results from a recent meta-analysis that assessed five randomized controlled trials on MSC-based therapy for knee OA [64,67,78,79,81] found that limited evidence for pain relief and functional outcomes does exist, yet there is a lack of evidence to support the claim that MSC-based therapy facilitates cartilage repair [115].

## 8. Cell Dose

The evaluation of cell dosing is important in order to find the minimum dose of MSCs, which is safe and still provides best overall outcomes. Given the limited amount of data and protocols on MSC therapy, most of the included studies were in fact dose-finding phase I or II trials.

The used cell doses ranged from the lowest mean dose of 1 × 10^6^ autologous BMMSCs [72] to a mean dose of 1.5 × 10^8^ allogenic BMMSCs [78]. The lowest mean dose was used by Chahal et al. in a dose-finding trial from 2019. Twelve knee OA patients were treated with escalating cell doses. Best clinical and radiological results were obtained in the high dose group of 5 × 10^7^ cells [72].

Gupta et al. compared four doses of allogenic BMMSCs (2.5 × 10^7^, 5 × 10^7^, 7.5 × 10^7^ and 1.5 × 10^8^ BMMSCs mixed with 2 or 4 mL of PLASMA-LYTE A followed by an injection of HA (20 mg) in 60 knee OA patients in a randomized, double-blinded, multicentric, placebo-controlled study. They were able to show that the lowest dose of 2.5 × 10^7^ cells provided the maximum pain reduction in all subjective parameters (WOMAC and pain VAS score), yet the observed improvement was not significant, when compared to placebo [78].

Pers et al. compared three cell doses (2 × 10^6^, 1 × 10^7^ and 5 × 10^7^ autologous ATMSCs in the form of SVF) and showed that patients treated with the lowest dose experienced significant improvements in pain and function WOMAC sub-scores, compared to baseline values. Of note, patients in the low-dose group had higher pain and function WOMAC sub-scores at baseline, compared to those receiving higher doses, which may have put a bias on the reported results [101].

In contrast, other authors yielded the best results for much higher cell doses in their studies [64,99,100,114]. Jo et al. compared three doses of autologous ATMSCs (1 × 10^7^, 5 × 10^7^ and 1 × 10^8^ ATMSCs) for the treatment of 18 knee OA patients. Improvements in the WOMAC score and improved cartilage volumes in MRI were observed in the high-dose group after six months. Second-look arthroscopy revealed a significant reduction of cartilage defects and a reduction of the International Cartilage Research Society (ICRS) grade in the high-dose group [99].

Jo et al. continued evaluating patients in a second phase of the study for a two-year follow-up. Improvements in WOMAC score, the Knee Society Score (KSS), the Knee injury and Osteoarthritis Outcome Score (KOOS) and reduced knee pain for up to 24 months were observed in both, the low- and the high-dose group. However, statistical significance was reached mainly in the high-dose group. Clinical outcomes and MRI scores declined after 12 months in the low- and mid-dose group, whereas a plateau was observed in the high-dose group until the 24-month follow-up [100]. A 2019 study by Freitag et al. found no differences between patients who were treated with a single injection of 1 × 10^8^ cells or those who were treated with repeated injections of the same dose, six months apart (each time 1 × 10^8^ cells) [83].

A retrospective analysis of 373 patients, who received autologous BMAC and PRP for 424 osteoarthritic knee joints, was able to demonstrate that patients receiving > 4 × 10^8^ cells showed significantly lower post-treatment numeric pain scale values than patients receiving < 4 × 10^8^ cells. However, improved function according to the Lower Extremity Functional Scale and the International Knee Documentation Committee (IKDC) score were seen in all patients, regardless of dose. The preference of the relatively high cell dose in this study was biased by the cell count, which included all nucleated cells in the BMAC (BMMSCs, haematopoietic stem cells, monocyte precursor cells, macrophages, B- and T-cells, etc.) and not only MSCs [117].

The analysis of the existing data underlines that only limited evidence exists on the most efficient cell dose in OA treatment to date. The range of the ideal therapeutic doses is still open for debate and discussed vividly among physicians and scientists. Yet, data shows that a dose range from a mean of 1 x 10^6^ autologous BMMSCs [72] to a mean of 1.5 × 10^8^ allogenic BMMSCs [78] appears to be safe and effective in selected parameters.

Finally, a meta-analysis by Kim et. al. could not find a recommended cell dose, due to a great variation of concentrations, applied in different randomized controlled trials [115].

Further dose-finding, randomized, controlled, blinded and preferably matched trials with larger numbers of participants are needed in order to avoid biased results.

## 9. Tissue Origin

The majority of trials covered by this review used autologous ATMSCs for OA treatment. Twelve studies used ATMSCs in the form of SVF [81,82,85,86,88,89,90,91,93,94,96,101], ten studies used pure-expanded autologous ATMSCs (including one follow-up study) [80,83,84,87,92,97,98,99,100,102] and one study used concentrated adipose tissue [95]. Fourteen studies used autologous BMMSCs (including two follow-up studies) [64,65,66,67,68,69,70,71,72,73,74,75,76], two used allogenic BMMSCs [78,79], two allogenic umbilical cord-derived MSCs (UCMSCs) [103,104] and one study used allogenic placenta-derived MSCs (PLMSCs) [105].

ATMSCs can easily be harvested through a simple and minimally invasive liposuction procedure. Liposuctions can be performed repeatedly and harvested cells show rapid expansion potential when cultured [118]. Adipose tissue contains a substantially higher number of MSCs than bone-marrow and ATMSCs are less affected by age and morbidity of patients. Further, ATMSCs maintain their differentiation potential even at later stages of life [119]. ATMSCs also exhibit the strongest anti-inflammatory potential when compared with other MSC sources [120].

Numerous clinical studies have proven the safety and efficacy of SVF therapy for OA. The isolation process and injection can usually be performed during the same visit, which makes SVF treatment an attractive alternative to pure ATMSC injections. Despite these advantages, the use of autologous ATMSCs has limitations. Cell manipulation due to ex vivo preparation and a lack of standardized harvesting protocols may impact the quality and quantity of cells. In this context, the MSC secretome, a valuable source of EVs, growth factors, and cytokines, has emerged as a possible cell-free therapeutic alternative [121]. Tofiño-Vian et al. were able to show that ATMSCs could serve as a source for EV exploitation [122], yet OA treatment, using the isolated secretome, has so far only been conducted in pre-clinical studies [121,123].

Currently recruiting clinical trials (see Figure 1) show a growing trend towards the use of allogenic UCMSCs and Wharton Jelly-derived MSCs (WJMSCs). Park et al. used UCMSCs in the treatment of seven knee OA patients and performed an extended follow-up of seven years. The study reported improved cartilage tissue at the 12-week follow-up and significantly improved pain VAS and IKDC scores at the 24-week follow-up. Improved clinical scores remained stable without significant deterioration for up to seven years. MRI findings showed preserved regenerated cartilage after three years [104]. A recently published study supports the favourable safety and efficacy profile of UCMSCs for knee OA treatment in 23 patients [103]. PLMSCs were also reported to be safe and effective regarding pain relief and improved range of motion in OA patients. Significant changes in the PLMSC-treated group were however only detected until eight weeks after treatment, when compared to the saline-treated control group [105]. Further studies with bigger patient samples are required to assess the safety and efficacy of UCMSCs and PLMSCs in depth.

Direct comparisons of MSCs from different tissue origins are scarce, yet Mautner et al. compared the efficacy of BMAC with that of microfragmented adipose tissue for the treatment of knee OA. Both autologous treatments significantly improved pain and function scores over time, yet no significant difference was seen between the two groups [71]. Vega et al. conducted a direct efficacy comparison between their own study from 2015 [79] using allogenic BMMSCs and a study by Orozco et al. from 2013 [76] using autologous BMMSCs and a study by Jo et al. from 2014 [99] using autologous ATMSCs for the treatment of knee OA.

For the inter-study comparison, the group evaluated the efficacy by taking pain relief divided by the initial pain score, a method described by Huskisson et al. in 1974 to provide a tool for assessing general treatment efficacy [124]. The results showed the best efficacy of 0.75 for the autologous BMMSC treatment (Orozco et al. [76]) with an effect size of 1.29 versus an efficacy of 0.39 and an effect size of 0.96 for the autologous ATMSC treatment (Jo et al. [99]). A slightly worse efficacy of 0.36 was determined for the allogenic BMMSC treatment with an effect size of 1.07 (Vega et al. [79]).

Due to the differing patient numbers, control arms, follow-ups and injection protocols of MSC injections, a comparison of studies like this has limited relevance. This was also concluded by Shariatzadeh et al. in a recent review comparing the level of efficiency of different MSC sources for knee OA treatment. The authors concluded that exact protocols of MSC characteristics, culture, dosage, and clinical application are necessary for a final evaluation and efficient comparison of different studies [39].

Vega at al. suggested future studies that directly compare autologous and allogenic MSCs within the same study [79]. Applying similar treatment protocols to both study arms would help to determine the most suitable source of MSCs, and analogous to dose-finding trials, “source-finding trials” would improve knowledge on safety and efficacy of different MSC tissue origins.

## 10. Independent Outcome Predictors

Identifying factors that are directly associated with clinical outcomes of a treatment is valuable as it allows prognostic statements about the efficacy of a potential therapy. Schiavone Panni et al. could recently demonstrate that patients with a baseline pain VAS score > 8 showed significantly greater improvements in clinical and functional outcomes after SVF-based treatment, when compared to patients with pain VAS scores < 8 [90].

Koh et al. observed that overweight patients (BMI > 27.5 kg/m^2^) and patients with a large cartilage lesion size (≥5.4 cm^2^) showed significantly worse clinical (IKDC score and Tegner activity scale) and arthroscopic (ICRS grade) outcomes in ATMSC-treated knee OA patients. Other factors, including age and sex, did not have a significant influence on outcomes [92]. The negative effect of morbid obesity on ATMSCs’ proliferation potential and multilineage differentiation capacity was proven in previous animal and human studies and may be linked to the high secretion of inflammatory cytokines in overweight patients [125,126].

Kim et al. [91] confirmed these findings in a retrospective study from 2015, analysing 49 knee OA patients after intra-articular autologous ATMSC treatment. The group could show that patient age and cartilage lesion size were independent predictors of clinical failure. Patients older than 60 years and patients with cartilage lesions larger than 6.0 cm^2^ were at risk of poor clinical outcomes (IKDC score and Tegner activity scale). The study also showed a statistically significant association between age and cartilage lesion size as well as between BMI and cartilage lesion size. Sex, side of involvement (left or right), lesion location and BMI could not predict clinical outcomes independently [91].

These findings were contradicted by a 2016 study from the same group, treating 20 knee OA patients with an arthroscopic autologous ATMSC implantation. The study concluded that sex, BMI, size and location of the cartilage lesion were no independent risk factors for poor clinical outcomes [94].

Soler et al. described patients younger than 65 years with mild to moderate OA of the knee as the ideal candidates for intra-articular MSC application. In their trial from 2016, the group treated 15 patients with a mean age of 52 years with autologous BMMSCs and showed significant improvements in clinical scores and cartilage repair (MRI) after 12 months [74].

Koh et al. examined 30 knee OA patients, older than 65 years of age, who received intra-articular autologous ATMSC injections in 2015. Almost all patients showed significant improvements in KOOS, pain VAS and Lysholm scores at the final two-year follow-up. Cartilage status improved or was maintained in 87.5% of the patients two years after treatment and none of the patients underwent total knee arthroplasty during the two-year follow-up period [93]. These findings question the cut off for intra-articular MSC therapy at the age of 60, suggested by Kim at al. [91]. Finally, Chahal et al. showed that anti-inflammatory markers, assessed from autologous BMMSCs after harvesting, were strongly predictive of clinical outcomes, including WOMAC and KOOS scores [72]. This points towards potential valuable screening panels, that may be applied in the future. Such panels could help identify patients, who would benefit most from MSC-based treatment, and those who would not.

## 11. Conclusions and Outlook

MSC therapies have been successfully realized in a limited number of clinical trials, treating patients with OA. First impressions show that locally applied MSCs seem to halt disease progression, partially regenerate cartilage and alleviate pain. Yet, the existing clinical studies vary strongly in treatment protocols, levels of evidence and follow-up periods. Interpretation of these data demands a differentiated view and more scientific engagement is undoubtedly required to provide further evidence for the specific efficacy of MSC treatment in OA. Thus, we suggest keeping a substantial critical view on these innovative new therapeutics and propose to establish standards in documentation, clinical trial management and handling to ensure comparability across trials.

The vast majority of MSC studies for OA target the knee joint, most likely due to the high prevalence amongst all forms of OA. Yet, results from included studies do not allow a direct translation to other joints. Shoulder and hip joints for example have different biomechanics compared to the knee joint and therefore have to be analysed from that view. Whereas we are hoping for more randomized, double-blinded, multicentric, placebo-controlled phase III trials in knee OA, there is also a big need for phase I and II studies, examining other forms of OA.

According to the trials included in this review, MSC treatment for knee OA has shown only a few adverse effects, which were all likely due to the procedural nature of intra-articular injections and MSC harvesting procedures. However, the majority of safety studies provided only a short follow-up period or a limited sample size, and thus final safety conclusions remain difficult. Results indicate some efficacy in selected parameters over a duration of at least several months. The biggest drawback of the current clinical evidence for MSC-based therapy is that most of the included studies did not have resilient control groups, which reduces the level of evidence and makes results less reliable. Therefore, a detailed analysis and comparison of the costs and therapeutic alternatives for the daily clinical practice seem necessary. Most clinical trials include patients with mild to moderate OA, where MSCs are believed to work best by targeting the persisting low-grade inflammation. Patients with more severe OA proved not to be the ideal candidates for MSC-based treatments, as osteophytes, subchondral sclerosis and cysts are unlikely to be altered by MSCs. Therefore, MSCs cannot be understood as an alternative to arthroplasty surgery, but potentially as an additional therapy for early onset and mild to moderate cases of OA.

Although the mechanisms by which MSCs exert their effects on affected joints are not fully understood, it is generally accepted that immunomodulatory and anti-inflammatory properties on the one hand and repair and restoration mechanisms on the other hand, have a synergistic or additive effect on the osteoarthritic joint. Future research should put a critical focus on the emerging role and potential clinical application of the MSC secretome and its biologically active factors. The enhancement and modulation of MSCs’ paracrine effects by priming may further increase their capacities to adequately alleviate pain and potentially restore joint integrity and function in OA-troubled patients.

## Figures and Tables

**Table 1:** Studies “currently recruiting” patients for MSC-based therapies for OA, listed on clinicaltrials.gov, categorized by study design and sorted by number of patients.

Indication	Number of Patients	Source of MSCs	Intervention	Control	Study Phase	Location and Study Identifier
**Double-blinded randomized controlled trials**						
Knee OA	260	Autologous ATMSCs	Single intra-articular injection of ATMSCs (10^8^ cells) vs. control	Saline	III	Cheongju-si, Daegu, Jeonju, Jinju-si, Seoul, all: Republic of Korea (South) (NCT03990805)
Knee OA	153	Autologous ATMSCs	Single intra-articular injection of ATMSCs (1 or 2 × 10^7^ cells) vs. control	Saline	II	Montpellier, France (NCT02838069)
Knee OA	60	Allogenic UCMSCs	Single intra-articular injection of allogenic UCMSCs (10^7^ cells) + PRP (5 mL) vs. control	HA	I/II	Liaocheng, Shandong, China (NCT03166865)
Knee OA	60	Autologous BMMSCs	Repeated (three) intra-articular injections of autologous BMMSCs + PRP (3 mL) vs. control over six months	PRP	II	Yantai, Shandong, China (NCT03969680)
Knee OA	40	Autologous ATMSCs	Single intra-articular injection of autologous ATMSCs vs. control	GC	III	Stanford, California, USA (NCT03467919)
Knee OA	24	BMMSCs	Single intra-articular injection of BMMSCs (10^8^ cells) vs. control	Saline	I/II	Seoul, Republic of Korea (South) (NCT04240873)
**Randomized controlled trials**						
Knee OA	60	Dental pulp mesenchymal stem cells	Single intra-articular injection of low dose dental pulp MSCs vs. high dose dental pulp MSCs vs. control	HA	I	Shanghai, China (NCT04130100)
Knee OA	60	Autologous ATMSCs	Repeated (three) intra-articular injections of autologous ATMSCs + PRP (3 mL) vs. control over six months	PRP	II	Yantai, Shandong, China (NCT04212728)
Knee OA	48	Autologous ATMSCs	Single intra-articular injection of autologous ATMSCs vs. control	GC	III	Beirut, Lebanon (NCT04230902)
Knee OA	30	Allogeneic ATMSCs	Single intra-articular injection of allogeneic ATMSCs (6.7 × 10^6^ or 4 × 10^7^) vs. control	HA	I/II	Hsinchu, Taiwan (NCT03943576)
Knee OA	30	Autologous ATMSCs	HTO followed by intra-articular autologous ATMSC injection vs. control, one month post osteotomy	HA	I/II	Jinan, Shandong, China (NCT03955497)
Knee OA	9	Allogenic UCMSCs	Repeated (three) intra-articular injections of allogenic UCMSCs (10^7^ cells) + HA (2 mL) + recombinant human somatropin (8 IU) vs. allogenic UCMSCs + HA vs. control weekly over three weeks	HA	I	Jakarta, Indonesia (NCT03800810)
**Prospective uncontrolled trials**						
Knee, hip, glenohumeral OA	100	Autologous ATMSCs	Repeated (three) intra-articular injections of autologous ATMSCs every three months over 12 months (≥ 10^6^ cells per injection)	-	I/II	Warsaw, Poland (NCT03869229)
Knee, hip, glenohumeral OA	100	Allogenic WJMSCs	Repeated (three) intra-articular injections of allogenic WJMSCs every three months over 12 months (≥ 10^6^ cells per injection)	-	I/II	Warsaw, Poland (NCT03866330)
Glenohumeral OA	30	Autologous BMAC	Single intra-articular injection of autologous BMAC (9 mL)	-	-	Rozzano, Milano, Italy (NCT04308213)
Knee OA	25	Autologous BMAC	Single subchondral and intra-articular injection of autologous BMAC (6 mL)	-	-	Rozzano, Milano, Italy (NCT03790189)
Hip OA	24	Autologous ATMSCs	Single intra-articular injection of ATMSCs (3 × 10^7^ cells) vs. two injections with one-month between injections	-	I	Rochester, Minnesota, United States (NCT03608579)
Knee OA	24	Allogenic UCMSCs	Single intra-articular injection of allogenic UCMSCs (2, 20 or 80 × 10^6^ cells)	-	I	Santiago, Chile (NCT03810521)
Knee OA	24	Autologous ATMSCs	Single or monthly repeated (three) intra-articular injection(s) of autologous ATMSCs (5 or 10 × 10^7^ cells)	-	I	Rochester, Minnesota, United States (NCT02805855)
Knee OA vs. focal chondral defect of the knee	16	Autologous BMMSCs	Single intra-articular injection of autologous BMMSCs (5 × 10^7^ cells)	-	I	Cleveland, Ohio, United States (NCT03477942)
Knee OA	15	Allogenic BMMSCs	Single intra-articular injection of allogenic BMMSCs (1, 5 or 10 × 10^7^ cells)	-	I/IIa	Taipei, Taiwan (NCT03589287)
Knee OA	12	Allogenic UCMSCs	Single intra-articular injection: low dose vs. mid dose vs. high dose (4, 10 or 20 × 10^6^ cells / 2 mL)	-	I	Seoul, Jongno-gu, Republic of Korea (South) (NCT04037345)
Knee OA	10	Allogenic WJMSCs	Repeated (two) intra-articular injections of allogenic WJMSCs (5 × 10^7^ cells in each dose)	-	I	Amman, Jordan (NCT02963727)
Knee OA	10	Autologous ATMSCs	Repeated (two) intra-articular injections of autologous ATMSCs (5 × 10^7^ cells in each dose)	-	I	Amman, Jordan (NCT02966951)
Knee OA	8	Autologous ATMSCs	Single intra-articular injection of ATMSCs	-	-	Qilu, Shandong, China (NCT03956719)

ATMSC = adipose tissue-derived mesenchymal stromal cell; BMAC = bone-marrow aspirate concentrate; BMMSC = bone-marrow-derived mesenchymal stromal cell; GC = glucocorticoid; HA = hyaluronic acid; HTO = high tibial osteotomy; MSC = mesenchymal stromal cell; OA = osteoarthritis; PRP = platelet-rich plasma; UCMSC = umbilical cord-derived mesenchymal stromal cell; WJMSC = Wharton Jelly-derived mesenchymal stromal cell.

**Table 2:** Included studies, categorized by tissue origin and sorted by study type, number of patients and follow-up.

References	OA Site	Severity of OA	Number of Patients	Follow-Up	Blin-Ding	Details of Treatment Arms and Mean Cell Numbers	Outcome Measures	Study Type	Control Arm	Main Results
	Autologous BMMSCs/BMAC									
Lamo-Espinosa et al., Journal of Translational Medicine (2016) [64]	Knee	K.L. ≥ 2	30	12 months	Double-blinded	Intra-articular injection of 10^7^ cells (*n* = 10) mixed with 1.5 mL of saline vs. 10^8^ cells (*n* = 10) mixed with of saline, both followed by an injection of 4 mL of HA (60 mg) vs. control	Pain VAS score, WOMAC score, range of motion, x-ray, MRI (WORMS)	Randomized, double-blinded, multicenter, placebo-controlled trial	Intra-articular injection of of HA (60 mg) (*n* = 10)	- No treatment-related SAEs or AEs. - Pain VAS score improved significantly in low- and high-dose treatment groups compared to baseline over time. WOMAC score improved significantly in the high-dose treatment group compared to baseline over time. The range of motion increased significantly in both treatment groups compared to baseline. X-ray images revealed joint space reduction in the control group but not in the high-dose treatment group (assessment in the low-dose group was not possible). MRI improvements were only seen in the high-dose treatment group.
Lamo-Espinosa et al., Journal of Translational Medicine (2018) [65]	“	“	27	48 months	“	“	Pain VAS score, WOMAC score	Follow-up visit to Lamo-Espinosa et al., Journal of Translational Medicine (2016) [64]	“	- No SAEs or AEs. - At the 4-year follow-up, significant improvements in pain VAS and WOMAC scores were observed for the low- and high dose group compared to the control group, which showed a progressive deterioration.
Emadedin et al., Cytotherapy (2018) [66]	Knee	K.L. 2–4	43	Six months	Triple-blinded	Intra-articular injection of 4 × 10^7^ cells in 5 mL of saline with 2% human serum albumin (*n* = 19) vs. control	Pain VAS score, WOMAC score, walking distance, standing time, range of motion	Randomized, triple-blinded, placebo-controlled trial	Intra-articular injection of of saline with 2% human serum albumin (*n* = 24)	- No significant AEs. - Significant improvement in painless walking distance and WOMAC pain sub-scale in the MSC group compared to placebo over time.
Wong et al., Arthroscopy (12/2013) [67]	Knee	-	56	24 months	-	HTO, microfracturing and intra-articular injection of 1,46 × 10^7^ cells mixed with 2 mL of HA (*n* = 28) vs. control	IKDC score, Tegner activity scale, Lysholm score, MRI (MOCART score)	Randomized controlled trial	HTO, microfracturing and intra-articular injection of 2 mL of HA (*n* = 28)	- No treatment-related SAEs. - The MSC group showed a significantly greater added improvement in all clinical scores, compared to the control group at the final follow-up. MRI showed significantly better results compared to the control group after one year.
Varma et al., Journal of the Indian Medical Association (2010) [68]	Knee	-	50	Six months	Double-blinded	Intra-articular injection of BMAC alongside arthroscopic debridement (*n* = 25) vs. control	Pain VAS score, KOOS	Randomized controlled trial	Arthroscopic debridement (n = 25)	- The MSC group showed a continuous decline of pain VAS score and KOOS at each follow-up compared to the control group.
Bastos et al., Knee Surgery, Sports Traumatology, Arthroscopy (2019) [69]	Knee	-	47	12 months	Double-blinded	Intra-articular injection of MSCs alone (*n* = 16) vs. MSCs + PRP (*n* = 14) vs. control	KOOS, range of motion, intra-articular cytokines	Randomized controlled trial	Intra-articular injection of GC (4 mg of dexamethasone) (*n* = 17)	- Significant improvement in all three groups in most KOOS domains and global score after one month and in all KOOS domains and global score at the 12-month follow-up. MSC and MSC + PRP groups showed the highest percentage of improvement.
Bastos et al., Knee Surgery, Sports Traumatology, Arthroscopy (2018) [70]	Knee	Dejour grade I–IV	18	12 months	Double-blinded	Intra-articular injection of MSCs + PRP (*n* = 9) vs. injection of MSCs alone (*n* = 9)	KOOS	Prospective randomized cohort study	-	- Two treatment-related SAEs (intense knee pain) and eight treatment-related AEs that resolved without any sequelae in five patients. - KOOS was significantly improved in both groups throughout a follow-up period of 12 months. No differences in clinical outcomes were observed between groups.
Mautner et al., Stem Cells Translational Medicine (2019) [71]	Knee	K.L. 1–4	76	13–22 months	-	Intra-articular injection of BMAC (*n* = 41) vs. micro fragmented adipose tissue (*n* = 35)	Pain VAS score, KOOS, EQOL,	Retrospective comparative study	-	- Significant improvement in KOOS, EQOL, and pain VAS score for both groups with no significant difference between groups.
Chahal et al., Stem Cells Translational Medicine (2019) [72]	Knee	K.L. ≥ 3	12	24 months	Radiologist blinded	Four cohorts; *n* = 3 in each of the first three cohorts received 1, 10 or 50 × 10^6^ cells; in cohort four *n* = 3 received either 1, 10 or 50 × 10^6^ cells	WOMAC score, KOOS, MRI (WORMS)	Prospective, uncontrolled trial	-	- No SAEs, four patients with minor transient AEs. - All four patients in the 50 × 10^6^ group achieved the minimal important difference (MID) in all scores except for KOOS (2/4 patients showed improvements) and quality of life (3/4) at the 12-month follow up; two of four patients in the 1 x 10^6^ group and one in the 10 × 10^6^ group showed MID in all scores; the 50 × 10^6^ cohort had the greatest number of patients achieving MID At higher MSC doses cartilage catabolic biomarkers and MRI-detected synovitis were significantly lower.
Emadedin et al., Archives of Iranian Medicine (2015) [73]	Knee (n = 6), ankle (n = 6), hip (n = 5)	K.L. 3/4	17	30 months	-	Intra-articular injection of 5 × 10^5^ cells/kg bodyweight	Pain VAS score, WOMAC score, HHS, FAOS, walking distance, MRI (T2)	Prospective, uncontrolled trial	-	- No treatment-related SAEs and a limited number of local treatment-related AEs. - Significant improvements in WOMAC score and walking distance in all patients. Pain VAS score improved significantly at the six- and 12-month follow-up and increased again afterwards. Radiological improvements were mainly present in the first six months (descriptive only).
Soler et al., The Knee (2016) [74]	Knee	K.L. 2/3	15	12 months	-	Intra-articular injection of 40.9 × 10^6^ cells (XCEL-M-ALPHA)	Pain VAS score, WOMAC score, Lequesne index, MRI (T2), aHAQ, SF-36	Prospective, uncontrolled trial	-	- No treatment-related SAEs and 16 musculoskeletal and connective tissue AEs. - Significant improvement in pain VAS and WOMAC scores, aHAQ and Lequesne index were seen over time. No significant changes were observed regarding the social and emotional roles and the mental health part of the SF-36. MRI showed significant improvements (cartilage regeneration) over time.
Al-Najar et al., Journal of Orthopaedic Surgery and Research (2017) [75]	Knee	K.L. 2/3	13	24 months	-	Two intra-articular injections one month apart, totalling 6.1 × 10^7^ ± 0.6 × 10^6^ cells	KOOS, MRI (T2)	Prospective, uncontrolled trial	-	- No treatment-related SAEs. - Significant improvement of cartilage thickness at the 12-month follow up (MRI) and significant improvement of KOOS at six, 12 and 24 months.
Orozco et al., Transplantation (2013) [76]	Knee	K.L. 2–4	12	12 months	-	Intra-articular injection of 4 × 10^7^ cells	Pain VAS score, WOMAC score, Lequesne index, MRI (T2, PCI), SF-36	Prospective, uncontrolled trial	-	- No treatment-related SAEs and a few minor transient treatment-related AEs. - Significant improvement of pain VAS and WOMAC scores and Lequesne index over time. No significant changes in SF-36. MRI revealed a significant decrease in PCI at six and 12 months. A greater and faster pain relief was noted during sports performance.
Orozco et al., Transplantation (2014) [77]	“	“	“	24 months	“	“	Pain VAS score, WOMAC score, Lequesne index, MRI (T2, PCI), SF-36	Follow-up visit to Orozco et al., Transplantation (2013) [76]	“	- No treatment-related SAEs and AEs. - Pain improvement was maintained. Further significant cartilage improvements were seen in MRI (PCI) compared to baseline at the 24-month follow-up.
	Allogenic BMMSCs									
Gupta et al., Arthritis Research and Therapy Journal (2016) [78]	Knee	K.L. 2/3	60	12 months	Double-blinded	Intra-articular injection of 2.5 × 10^7^ cells (*n* = 10) vs. 5 × 10^7^ cells (*n* = 10) vs. 7.5 × 10^7^ cells (*n* = 10) vs. 15 × 10^7^ cells (*n* = 10) (Stempeucel^®^) mixed with 2–4 mL PLASMA-LYTE A followed by a 2 mL injection of HA (20 mg) vs. control	Pain VAS score, WOMAC score, ICOAP, MRI (WORMS)	Randomized, double-blinded, multicentric, placebo-controlled trial	Intra-articular injection of 2–4 mL of PLASMA-LYTE A (*n* = 20)	- One treatment-related SAE (synovial effusion) in the 150 × 10^6^ cells group and nine treatment-related AEs among all cell-dose groups. - A decrease of pain VAS score and ICOAP in all treatment groups, except for patients who received 15 × 10^7^ cells, over time, yet without significance when compared to placebo. WOMAC score decreased in all treatment groups, again without significance. Although not significant, the maximum pain reduction in all scores was observed in the low-dose group. No MRI changes were described.
Vega et al., Transplantation (2015) [79]	Knee	K.L. ≥ 2	30	12 months	Double-blinded	Intra-articular injection of 4 × 10^7^ cells (*n* = 15) vs. control	Pain VAS score, WOMAC score, Lequesne index, MRI (T2, PCI), SF-12	Randomized controlled trial	Intra-articular injection of 3 mL of HA (60 mg) (*n* = 15)	- No SAEs and few mild transient AEs, that affected both groups. - Significant pain reduction in the treatment group at six- and 12-month follow-ups (pain VAS score, WOMAC score, Lequesne index). The active control showed a significant improvement in pain VAS score only at the 12-month follow-up. The treatment group showed significant MRI improvements at the 12-month follow-up.
	Autologous ATMSCs									
Lee et al., Stem Cells Translational Medicine (2019) [80]	Knee	K.L. ≥ 2	24	Six months	Double-blinded	Intra-articular injection of 10^8^ cells (*n* = 12) vs. control	WOMAC score	Randomized, double-blinded, placebo-controlled trial	Intra-articular injection of saline (*n* = 12)	- MSC group with significant improvement of WOMAC score at six months; control group without significant changes in WOMAC score at the same follow-up.
Koh et al., Arthroscopy (2014) [81]	Knee	K.L. ≤ 3	44	24–25 months	Single-blinded	HTO and intra-articular injection of 4.11 × 10^6^ cells (SVF) mixed with of PRP (*n* = 21) vs. control	Pain VAS score, KOOS, Lysholm score, Kanamiya grading (arthroscopic)	Randomized controlled trial	HTO and intra-articular injection of 3 mL of PRP (*n* = 23)	- Significantly greater improvements in KOOS sub-scales and pain VAS score in the HTO + MSC + PRP group compared to the HTO + PRP group. Lysholm score was significantly improved in both groups at the final follow-up. Second-look arthroscopy findings after a mean of 19.8 months showed significantly better cartilage healing in the HTO + MSC + PRP group.
Hong et al., International Orthopaedics (2019) [82]	Knee	K.L. 2/3	16	12 months	Double-blinded	Arthroscopic implantation of SVF into one joint (*n* = 16) vs. control implantation into the contralateral joint	Pain VAS score, WOMAC score, range of motion, MRI (WORMS, MOCART score)	Randomized controlled trial	Arthroscopic implantation of 4 mL of HA (*n* = 16)	- No SAEs, few local AEs that resolved within two weeks. - Pain and functional scores improved significantly over time in SVF-treated knees, whereas HA-treated knees worsened over time without statistical significance. Significantly improved WORMS and MOCART scores were also reported for SVF-treated knees compared to HA-treated controls.
Freitag et al., Regenerative Medicine (2019) [83]	Knee	K.L. 2/3	30	12 months	-	Single intra-articular injection of 10^8^ cells (*n* = 10) vs. repeated (two) injections of 10^8^ cells six months apart (*n* = 10) vs. control	WOMAC score, KOOS, numeric pain rating scale, MRI (MOAKS)	Randomized controlled trial	Conservative treatment (*n* = 10)	- No SAEs, few self-limiting mild and moderate AEs. - Significant improvements for both treatment groups in pain and clinical outcomes over time and compared to control. No difference between treatment groups. Cartilage improvements or no further cartilage loss in the repeated injection group seen in MRI.
Kim et al., Knee Surgery, Sports Traumatology, Arthroscopy (2020) [84]	Knee	K.L. ≥ 3	80	12–27 months	-	HTO and intra-articular injection of cells (*n* = 40) vs. HTO and cells with allogenic cartilage (*n* = 40)	KOOS, Lysholm score, Kanamiya grading (arthroscopic)	Prospective randomized cohort study	-	- Clinical outcomes improved significantly for both groups at second-look arthroscopy. From the second-look arthroscopy to the final follow-up only the MSC allogenic cartilage group improved. Kanamiya grading was significantly higher in the MSC group with allogenic cartilage than in the MSC group.
Kim et al., The American Journal of Sports Medicine (2014) [85]	Knee	K.L. 1/2	54	24–34 months	-	Arthroscopic implantation of 3.9 × 10^6^ cells (SVF) without scaffold (*n* = 37) vs. arthroscopic implantation of 3.9 × 10^6^ cells (SVF) mixed with fibrin glue as a scaffold. (*n* = 17)	IKDC score, Tegner activity scale, ICRS grade (arthroscopic)	Retrospective comparative study	-	- Significant improvements of IKDC score and Tegner activity scale in both groups after a mean of 12.3 months. Significantly improved arthroscopic ICRS grades in the implantation + scaffold group compared to the implantation without scaffold group with a significant correlation between clinical outcomes and ICRS grades. Significant predictors for poor clinical outcomes in the implantation without scaffold group were overweight (BMI > 25.5kg/m^2^) and large lesion size (>5.7cm^2^). A similar trend was observed in the implantation + scaffold group, yet without significance.
Kim and Koh, The American Journal of Sports Medicine (2018) [86]	Knee	K.L. 2/3	100	32 months	-	HTO and intra-articular injection of a mean of 4.26 × 10^6^ cells (SVF) (*n* = 50) vs. control	IKDC score, Lysholm score, x-ray, ICRS grade (arthroscopic)	Comparative matched-pair study	HTO (*n* = 50)	- Significant clinical improvements in the HTO + MSC group compared to the HTO group at the final follow-up. ICRS grades correlated with clinical findings. Radiological outcomes improved compared to pre-operative findings, yet they did not correlate with clinical findings.
Koh and Choi, The Knee (2012) [87]	Knee	K-L. < 4	50	12–18 months	-	Arthroscopic debridement accompanied by intra-articular injection of 1.89 × 10^6^ cells, mixed with 3 mL of PRP (*n* = 25) vs. control	Pain VAS score, Tegner activity scale, Lysholm score	Retrospective comparative matched-pair study	Arthroscopic debridement and intra-articular injection of 3 mL of PRP (*n* = 25)	- No SAEs, one patient experienced marked knee pain with swelling, following the injection which resolved spontaneously. Few patients reported slight knee pain in the first two or three days after the injection. - Significant improvements in pain VAS score, Tegner activity scale and Lysholm score at the final follow-up in both groups. No significant differences between the study and control group at the last follow-up.
Kim and Koh., Arthroscopy (2016) [88]	Ankle	-	49	24–34 months	-	Lateral sliding calcaneal osteotomy with bone-marrow stimulation and intra-articular injection of 4.1 × 10^6^ cells (SVF) (*n* = 26) vs. control	Pain VAS score, AOFAS score, x-ray, ICRS grade (arthroscopic)	Retrospective comparative study	Lateral sliding calcaneal osteotomy with bone-marrow stimulation (*n* = 23)	- Significant improvements in pain VAS and AOFAS scores for both groups at the final follow-up compared to preoperative findings. Clinical scores showed a significantly greater improvement in osteotomy + stimulation + injection group compared to the control group. ICRS grades correlated with clinical findings and showed significantly greater improvements in the osteotomy + stimulation + injection group compared to the control group at the final follow-up. X-ray imaging showed significant improvements in both groups at the final follow-up compared to preoperative findings with no significant difference between groups.
Kim et al., The American Journal of Sports Medicine (2015) [89]	Knee	K.L. 1/2	40	24–42 months	-	Arthroscopic injection of 4.07 × 10^6^ cells (SVF) mixed with PRP (*n* = 20) vs. arthroscopic implantation of 3.96 × 10^6^ cells (SVF) on a fibrin glue scaffold (*n* = 20)	IKDC score, Tegner activity scale, ICRS grade (arthroscopic)	Retrospective comparative matched-pair study	-	- Significant improvements of IKDC score and Tegner activity scale at the final follow-up in the implantation group with a significant difference between groups. A significant correlation between clinical outcomes and ICRS grading (second-look arthroscopy after a mean of 12.6 months) was seen, with significantly greater improvements of ICRS grading in the implantation group.
Schiavone Panni, International Orthopaedics (2019) [90]	Knee	K.L. < 3	52	24 months	-	Arthroscopic debridement followed by intra-articular injection of 10–15 mL of SVF	Pain VAS score, IKS score	Retrospective case series	-	- No SAEs, three AEs related to harvesting procedure. - Significant improvement of pain VAS and IKS score over time. Patients with a baseline pain VAS score > 8 showed greater clinical and functional improvements.
Kim et al., The American Journal of Sports Medicine (2015) [91]	Knee	K.L. 1/2	49	24–36 months	-	Arthroscopic implantation of 4.3 × 10^6^ cells (SVF) on a fibrin glue scaffold	IKDC score, Tegner activity scale	Retrospective case series	-	- Significant improvements of IKDC score and Tegner activity scale at the final follow-up. A high prognostic significance was observed regarding age and lesion size. 60 years and a lesion size of 6.0 cm^2^ were observed as an optimum cut-off for poor clinical outcomes.
Koh et al., The American Journal of Sports Medicine (2014) [92]	Knee	K.L. 1/2	35	24–34 months	-	Intra-articular injection of 3.8 × 10^6^ cells	IKDC score, Tegner activity scale, ICRS grade (arthroscopic)	Retrospective case series	-	- Significant improvements of IKDC score and Tegner activity scale at the final follow-up. Overweight patients (BMI >27.5 kg/m^2^) and patients with a large lesion size (>5.4 cm2) had a significantly worse outcome regarding IKDC score, Tegner activity scale and ICRS grade. Both, the Tegner activity scale and the IKDC score were negatively correlated with ICRS grades. Second-look arthroscopic surgery revealed healed chondral lesions after a mean of 12.7 months.
Koh et al., Knee Surgery, Sports Traumatology, Arthroscopy (2015) [93]	Knee	K.L. 2/3	30	24–26 months	-	Intra-articular injection of 4.04 × 10^6^ cells (SVF) mixed with 3 mL of PRP combined with arthroscopic lavage	Pain VAS score, KOOS, Lysholm score, x-ray, ICRS grade (arthroscopic)	Prospective, uncontrolled trial	-	- No major complications, three patients complained of slight knee pain. - Significantly improved clinical scores at the 24-month follow-up compared to the 12-month follow-up. Second-look arthroscopy revealed 18.7% very positive, 43.8% positive, 25% neutral and 12.5% negative cartilage healing results. Age and mean improvement in KOOS sub-scales were associated. K.L. grade 2 was associated with a higher Lysholm score improvement.
Kim et al., Osteoarthritis and Cartilage (2016) [94]	Knee	K.L. 1/2	20	24 months	-	Arthroscopic implantation of 4.4 × 10^6^ cells (SVF) on a fibrin glue scaffold	IKDC score, Tegner activity scale, MRI (MOAKS, MOCART score)	Prospective, uncontrolled trial	-	- Significant improvement of IKDC score and Tegner activity scale at the final follow-up. Significant improvements in MRI scores with a correlation between clinical and MRI outcomes at the final follow-up.
Roato et al., International Orthopaedics (2018) [95]	Knee	K.L. 1–3	20	18 months	-	Arthroscopic implantation of 35 mL of concentrated autologous adipose tissue	Pain VAS score, WOMAC score, MRI (T1, T2)	Prospective, uncontrolled trial	-	- No SAEs and few self-limiting AEs, two patients underwent arthroplasty before trial completion. - Significant pain VAS and WOMAC score improvements at all follow-ups. No MRI changes. Histological sections of MSC-treated knees (who underwent arthroplasty) showed new tissue formation (descriptive only).
Pintat et al., Journal of Vascular and Interventional Radiology (2017) [96]	Knee	-	19	12 months	-	Intra-articular injection of cells (SVF) mixed with PRP	WOMAC score, MRI (lesion grade, surface, T2)	Prospective, uncontrolled trial	-	- No complications reported. - Significantly improved WOMAC score at the six- and 12-month follow-up compared to baseline. No significant MRI differences
Koh et al., Arthroscopy (04/2013) [97]	Knee	K.L. 3/4	18	24–26 months	-	Intra-articular injection of 1.18 × 10^6^ cells mixed with 3 mL of PRP	Pain VAS score, WOMAC score, Lysholm score, MRI (WORMS)	Prospective, uncontrolled trial	-	- Significant reduced WOMAC score and improved Lysholm and pain VAS score wat the final follow-up. MRI score showed a significant improvement at final follow-up.
Song et al., Regenerative Medicine (2018) [98]	Knee	K.L. ≥ 2	18	22 months	-	Intra-articular injection of 1 × 10^7^ cells (*n* = 6) vs. 2 × 10^7^ cells (*n* = 6) vs. 5 × 10^7^cells (*n* = 6) into each knee-joint, followed by a third injection of 5 × 10^7^ cells after eleven months	WOMAC score, NRS-11, MRI (cartilage volume), SF-36	Prospective, uncontrolled trial	-	- No SAEs and few AEs occurred equally distributed among groups. - WOMAC score and the NRS-11 significantly improved over time. SF-36 showed a significant reduction only at the three-month follow-up for the low-dose group and at the 22-month follow-up for the middle-dose group. MRI showed a significant increase in cartilage volume over time, compared to baseline. The high-dose group showed the greatest improvements overall.
Jo et al., Stem Cells (2014) [99]	Knee	K.L. ≥ 2	18	Six months	-	Intra-articular injections of 1 × 10^7^ cells (*n* = 3) vs. 5 × 10^7^ cells (*n* = 3) vs. 1 × 10^8^ (*n* = 12) cells mixed with saline	Pain VAS score, WOMAC score, KOOS, KSS, x-ray, MRI (size and depth of cartilage defect), ICRS grade (arthroscopic), histology (safranin O, immunohistochemistry)	Prospective, uncontrolled trial	-	- No treatment-related SAEs or AEs. - An improvement of pain VAS and WOMAC score at the six-month follow-up in the high-dose group. KSS improved significantly in the low- and high-dose group. MRI (size of cartilage defect) showed a significant improvement in the high-dose group. Second look arthroscopy revealed a significant reduction o cartilage defects and of the ICRS grade in the high-dose group. Histology showed regeneration of articular cartilage defects (descriptive only).
Jo et al., American Journal of Sports Medicine (2017) [100]	“	“	“	24 months	“	“	Pain VAS score, WOMAC score, KOOS, KSS, x-ray, MRI (size and depth of cartilage defect)	Follow-up visit to Jo et al., Stem Cells (2014) [99]	“	- No treatment-related SAEs or AEs. - Improvement in WOMAC score, KSS, KOOS and reduced knee pain for up to 24 months were seen for any dose. Statistical significance was reached mainly in the high-dose group. Clinical outcomes (WOMAC score) declined after 12 months in the low- and mid-dose group, whereas a plateau was observed in the high-dose group until 24 months. Similar results were obtained for MRI evaluations.
Pers et al., Stem Cells Translational Medicine (2016) [101]	Knee	K.L. 3/4	18	Six months	-	Intra-articular injection of 2 × 10^6^ cells (SVF) (*n* = 6) vs. 10 × 10^6^ cells (SVF) (*n* = 6) vs. 50 × 10^6^ cells (SVF) (*n* = 6)	Pain VAS score, WOMAC score, KOOS, SAS, MRI (dGMERIC and T_1rho_), histology (protein S 100, CD 34, Ki 67) SF-36, PGA	Prospective, bicentric uncontrolled trial	-	- No treatment-related SAEs and five potentially treatment-related AEs. - Improvement in pain, function and mobility were observed, regardless of dose. Significantly improved pain levels and function were detected only in the low-dose group. No correlation between clinical changes and MRI. All but one patient refused a previously scheduled total knee arthroplasty. Histological findings varied in their description. No statistically significant differences between groups for SF-36.
Spasovski et al., The Journal of Gene Medicine [102]	Knee	IKDC B & D	nine	18 months	-	Intra-articular injection of 0.5–1 × 10^7^ cells	Pain VAS score, KSS, HSS-KS, Tegner-Lysholm score, X-ray, MRI (MOCART score)	Prospective, uncontrolled trial		- No SAEs and few AEs, that resolved within one week. - Significant improvements between baseline and 3-month follow-up and further improvements between 3- and 6-month follow-up in all scores. Scores remained improved at 12- and 18-month follow-up, without further significant development. A significant cartilage restoration (MOCART score) was observed at the final follow-up, compared to baseline. X-ray imaging showed neither improvement nor deterioration.
	Allogeneic UCMSCs									
Matas et al., Stem Cells Translational Medicine (2019) [103]	Knee	K.L. 1–3	26	12 months	Triple-blinded	Repeated (two) intra-articular injections of 2 × 10^7^ cells in 3 mL of saline with 5% plasma six months apart (*n* = 9) vs. single injection of 2 × 10^7^ cells (*n* = 9), followed by 3 mL of saline with 5% plasma six months apart vs. control	Pain VAS score, WOMAC score, SF-36, Patient Global Assessment, OMERACT-OARSI Responder Index Criteria, MRI (WORMS)	Randomized controlled trial	Intra-articular injection of 3 mL of HA (*n* = 8)	- No SAEs, few transient AEs (acute synovitis and local pain). - Significant pain and functional improvements over time for MSC-treated patients compared to control. The single injection group stopped improving after month 9, while the repeated injections group continued improving until final the follow-up. All patients in the repeated injections group were found to be responders, according to the OMERACT-OARSI Responder Index Criteria. No changes in SF-36 and MRI.
Park et al., Stem Cells Translational Medicine (2017) [104]	Knee	K.L. 3	seven	98 months	-	Implantation of “Cartistem”, a composite of UCMSCs and HA hydrogel, into drill holes at two different dosages: 1.15–1.25 × 10^7^ cells (*n* = 4) vs. 1.65–2 × 10^7^ cells (*n* = 3)	Pain VAS score, IKDC score, MRI (dGMERIC), ICRS grade (arthroscopic), histology (Masson’s trichrome, safranin O, immunohisto-chemistry)	Prospective, uncontrolled trial	-	- No treatment-related SAEs and one treatment-related AE. - Pain VAS and IKDC score improved significantly over time and remained improved without significant deterioration at the seven-year follow-up. MRI showed high glycosaminoglycan contents in regenerated cartilage (qualitative). Hyaline-like cartilage was found at lesion sites at the one-year arthroscopic follow-up in two patients with histological findings of regenerated cartilage (descriptive only).
	Allogenic PLMSCs									
Khalifeh Soltani et al., Cytotherapy (2019) [105]	Knee	K.L. 2–4	20	Six months	Double-blinded	Intra-articular injection of 5–6 × 10^7^ cells in 1mL of saline (*n* = 10) vs. control	KOOS, range of motion, MRI (cartilage thickness)	Randomized, double-blinded, placebo-controlled trial	Intra-articular injection of 10 mL of saline (*n* = 10)	- No SAEs and four self-limiting AEs. - Significantly improved pain score and range of motion in the MSC group at week 8. Cartilage thickness improved in the MSC group only.

AE = adverse event; ATMSC = adipose tissue-derived mesenchymal stromal cell; aHAQ = Algofunctional Health Assessment Questionnaire; AOFAS = American Orthopaedic Foot and Ankle Society; BMAC = bone-marrow aspirate concentrates; BMMSC = bone-marrow-derived mesenchymal stromal cell; dGMERIC = delayed gadolinium-enhanced-magnet resonance imaging of cartilage; EQOL = Emory Quality of Life; FAOS = Foot and Ankle Outcome Score; GC = Glucocorticoid; HA = hyaluronic acid; HHS = Harris Hip Score; HSS-KS = Hospital for Special Surgery knee score; HTO = high tibial osteotomy; ICOAP = Intermittent and Constant Osteoarthritis Pain Score; ICRS = International Cartilage Research Society; IKDC = International Knee Documentation Committee; IKS score = International Knee Society knee and function score; K.L. = Kellgren and Lawrence; KOOS = Knee injury and Osteoarthritis Outcome Score; KSS = Knee Society Score; MOAKS = MRI Osteoarthritis Knee Score; MOCART = Magnetic Resonance Observation of Cartilage Repair Tissue; MRI = magnetic resonance imaging; NRS-11 = numerical pain rating scale-11; OA = osteoarthritis; OAOS = Osteoarthritis Outcome Score; OMERACT-OARSI Responder Index Criteria = Outcome Measures in Rheumatology Committee (OMERACT)-Osteoarthritis Research Society International (OARSI) Responder Index Criteria; PCI = phase contrast imaging; PGA = Patient Global Assessment; PLMSCs = Placenta-derived mesenchymal stromal cells; PRP = platelet-rich plasma; SAE = serious adverse event; SAS = Short Arthritis Assessment Scale; SF-36 = Quality of Life Short Form-36; SF-12 = Quality of Life Short Form-12; SVF = stromal vascular fraction; UCMSC = umbilical cord-derived mesenchymal stromal cell; VAS = Visual Analogue Scale; WOMAC = Western Ontario and McMaster Universities OA Index; WORMS = Whole-Organ Magnetic Resonance Imaging Score. “ = follow up study.
